# Maturation of Eupyrene Sperm upon Ejaculation Is Influenced by a Male Accessory Gland-Derived Serine Protease in *Grapholita molesta*

**DOI:** 10.3390/insects16080782

**Published:** 2025-07-30

**Authors:** Jie Cheng, Tai Guo, Zhongyan Zhou, Wei Wei, Yu Liang, Huiming Xiang, Ruiyan Ma, Zhongjian Shen, Zhi-Guo Zhao

**Affiliations:** 1 College of Plant Protection, Shanxi Agricultural University, Taigu 030801, China; jiecheng1121@sxau.edu.cn (J.C.); a18397664495@163.com (T.G.); zzy13110079148@163.com (Z.Z.); 19581501921@163.com (W.W.); lllyyy222666@163.com (Y.L.); xianghuiming82@163.com (H.X.); maruiyan2019@163.com (R.M.); 2 Shanxi Key Laboratory of Bioagent Utilization and Eco-Pesticide Innovation, Taigu 030801, China; 3 State Key Laboratory for Biology of Plant Diseases and Insect Pests, Key Laboratory of Natural Enemy Insects of Ministry of Agriculture and Rural Affairs, Institute of Plant Protection, Chinese Academy of Agricultural Sciences, Beijing 100193, China

**Keywords:** oriental fruit moth, serine protease, male fertility, eupyrene sperm, spermatophore, metabolic signatures

## Abstract

*Grapholita molesta* is a globally significant fruit pest. Females achieve maximal reproductive output through efficient sperm utilization following a single copulation. Post-mating maturation of eupyrene sperm is a critical step in reproductive success. Here, we report that a male accessory gland-derived serine protease (named GmAGSP1) is essential for this process. GmAGSP1 was only distantly related to other identified sperm-activating SPs, and its transcript was highly expressed in the AG at 48 h after emergence. RNAi-mediated knockdown of *GmAGSP1* in males did not affect courtship rate, copulation duration, or mating frequency, whereas male fertility decreased significantly. Mating with *GmAGSP1*-knockdown males markedly impaired eupyrene sperm maturation in the spermatophores, with phenotypes including failure of eupyrene sperm bundles to dissociate normally and marked reduction in viability of the dissociated eupyrene sperm. Finally, untargeted metabolomic analysis preliminarily demonstrated marked alterations in multiple metabolic pathways within the spermatophore following mating with *GmAGSP1*-knockdown males. This study advances our understanding of the regulatory mechanism of “sperm activation in the spermatophore’s metabolic microenvironment mediated by male AG-derived SP” while providing critical insights for the development of novel genetic control strategies targeting *G. molesta*.

## Simple Summary

*Grapholita molesta* is a globally devastating fruit pest whose females achieve maximal fertility through single copulation-dependent eupyrene sperm maturation. This study identifies a male accessory gland-derived serine protease, GmAGSP1, as essential for post-mating sperm activation. RNAi-mediated depletion of *GmAGSP1* in males disrupts eupyrene sperm bundle dissociation and reduces eupyrene sperm viability within the spermatophore. Metabolomic profiling reveals marked alterations in spermatophore metabolic pathways, including dysregulation of pyruvate and TCA cycle metabolism, in matings with *GmAGSP1*-deficient males. These findings highlight GmAGSP1 as a potential target for developing genetic control strategies against this pest.

## 1. Introduction

In insect species, serine proteases (SPs) are a widespread family of hydrolytic enzymes that possess a catalytic triad (His, Asp, and Ser) [1]. These enzymes play crucial roles in various physiological processes, including food digestion, immune response, and signal transduction [2]. Intriguingly, SPs are frequently identified among seminal fluid proteins (SFPs) [3,4]. In lepidopterans, SPs are synthesized by the male accessory gland (AG) and classified as accessory gland proteins (Acps) [5]. These proteins, delivered to the female bursa copulatrix (BC, more specifically, spermatophore) during copulation together with two kinds of sperm (non-fertile apyrene sperm and fertile eupyrene sperm), have been determined to play crucial roles in reproductive success [6].

Over the past decade, a multitude of AG-derived SPs have been identified and characterized in lepidopterans [6,7,8,9]. However, only a few studies have demonstrated a direct role of SPs in sperm maturation. Working with *Bombyx mori*, Omura [10] first found that sperm maturation was driven by trypsin-like protease. Since then, investigators have determined that Initiatorin can stimulate apyrene sperm motility and dissociation of eupyrene sperm bundles (ESBs) [11]. In *Manduca sexta*, dissociation of ESBs and motility of both apyrene and eupyrene sperm have been observed as a direct consequence of in vitro culture with trypsin [12]. Recent studies have demonstrated that snake-like SP in *Spodoptera frugiperda* and trypsin-like SP in *Spodoptera litura* are also essential to sperm activation [13,14]. Although a few studies have suggested that SPs modulate the metabolism of small molecules in the spermatophore [11,13,15], the global metabolic profile mediated by SPs has not been comprehensively characterized. To date, only one metabolomic study has focused on the spermatophore [16], highlighting a significant gap in lepidopteran systems.

Here, we used the oriental fruit moth (OFM) *Grapholita molesta* (Lepidoptera: Tortricidae), a notorious Rosaceae fruit-boring pest worldwide, to study the function and metabolic signatures of SP during eupyrene sperm maturation in the spermatophore [17]. In this species, most females typically mate only once, and a single mating provides sufficient eupyrene sperm to fertilize mature eggs throughout the oviposition period [18]. After mating, eupyrene sperm maturation within the spermatophore is essential for reproductive success [19]. However, the functional characterization of SPs in this process, particularly concerning SPs’ roles at the metabolic level, is underexplored. Through transcriptomic analysis and subsequent comparative proteomic studies, we previously identified an AG-derived SP in *G. molesta* (named GmAGSP1) as a potential driver of eupyrene sperm maturation [17]. Accordingly, in this study, our goals were (i) to evaluate the reproductive role of *GmAGSP1* in vivo including sexual behaviors and fertility; (ii) to determine the phenotypic effects on eupyrene sperm maturation; (iii) to comprehensively identify the differential metabolites after *GmAGSP1* knockdown within the spermatophore. This study aims to characterize GmAGSP1, a putative sperm-activating serine protease, and evaluate its role in male fertility, sperm maturation, and associated metabolic changes.

## 2. Materials and Methods

### 2.1. Insects

The OFM was obtained from the Taigu Experimental Station, Institute of Pomology, and maintained in the Biosafety and Biocontrol Laboratory over ten generations (insecticide-free conditions, 25 ± 1 °C, 70% ± 10% relative humidity, 15:9 h light/dark photoperiod). Artificial diet and 10% honey water were added for rearing larvae and adults, respectively.

### 2.2. Cloning of GmAGSP1 Based on Quantitative Proteomic Data

GmAGSP1 was identified from the comparative proteomics (virgin AGs vs. mated AGs, ProteomeXchange, PXD063378) and then verified in the spermatophore proteomics (ProteomeXchange, PXD056714). The protein quantitative results showed that GmAGSP1 was identified as a typical Acp and was delivered to the female spermatophore after mating. The full-length nucleotide sequence of *GmAGSP1* (GenBank accession number: PQ364127) was acquired from AG RNA sequencing dataset of OFM (Genome Sequence Archive, CRA006004). The full-length cDNA sequence was cloned with corresponding primers for sequencing (Table S1). Other SP sequences were retrieved from the published articles. Phylogenetic analysis was conducted using the Neighbor-Joining method in MEGA v7.0, and multiple sequence alignments were generated with ClustalX. *Homo sapiens* trypsinogen (AAA61232.1) was selected as an outgroup.

### 2.3. qRT-PCR

Total RNA was extracted using the TRIzol reagent (TIANGEN, Beijing, China) from unmated sexually mature females, unmated sexually mature males, unmated sexually mature male tissues (head, thorax, fat body, gut, whole reproductive system, testis, and AG), and unmated male AGs at different growing stages (<2, 24, 48, 72, and 96 h post-eclosion). These RNA samples were reverse-transcribed using the PrimeScript RT reagent kit with gDNA Eraser (Takara Bio, Otsu, Japan). The TB Green™ Premix Ex Taq™ (Takara Bio) was used for qRT-PCR on the CFX Connect TM Real-Time PCR System (Bio-Rad, Hercules, CA, USA). The internal controls were the *Actin* (KF022227.1) and *Glyceraldehyde-3-phosphate dehydrogenase* (KJ094948.1) (Table S1), and the quantitative variation of GmAGSP1 was calculated using the 2^−ΔΔCT^ method.

### 2.4. RNA Interference

To avoid potential off-target effects, following previous research methods [20], a Python (version 3.12.2) script was used to cleave the coding sequence of *GmAGSP1* into all possible 19 bp small interfering RNAs (siRNAs) for BLAST (BLAST+, version 2.16.0) searches. The results indicated no risk of intraspecific off-target effects. Based on these findings, the Tryp_SPc domain of GmAGSP1 was used as the cDNA template, and dsRNAs were carried out using the T7 RiboMAX™ Express RNAi System (Promega, Madison, WI, USA) with primers attaching the T7 polymerase promoter (Table S1). After purification, the concentration, purity, and integrity of dsRNA were verified using a NanoDrop™ 2000 spectrophotometer and agarose gel electrophoresis. Newly emerged virgin OFM males (within 2 h) were injected with 5 μg of ds*GFP* (control) or ds*GmAGSP1* into the anterior thorax using a microinjector (Nanoject II, Drummond Scientific, Broomall, PA, USA). After 48 h, the knockdown efficiency of target gene in the AGs was measured using qRT-PCR. Then, the dsRNA-injected unmated sexually mature males were paired with age-matched virgin females for subsequent experiments.

### 2.5. Behavioral and Fertility Assay

The dsRNA-injected unmated sexually mature males were paired with untreated virgin sexually mature females in a transparent observation chamber (ds*GFP*♂ × untreated♀; ds*GmAGSP1*♂ × untreated♀). For sexual behavior tests, courtship and mating behaviors were recorded under infrared illumination during the scotophase using a night vision camera. Behavioral parameters were defined as previously described [17]. The proportion of males displaying courtship, the proportion of mating pairs, and the mating duration were recorded and then compared. For fertility assays, all successfully mated females were allowed to lay eggs for 7 consecutive days. The total number of eggs and the total number of hatched larvae were counted.

### 2.6. Determination of Eupyrene Sperm Morphology and Viability

Spermatophores from females mated with ds*GFP*♂ or ds*GmAGSP1*♂ males were dissected and avulsed. For morphological analysis of ESBs, ESBs were separated in PBS buffer according to the previous “panning method” [21]. The amount and dissociation state of ESBs at 180 min after mating were recorded under a microscope. For viability of eupyrene sperm, eupyrene sperm within spermatophores at 240 min after mating were collected in the physiological saline. Eupyrene sperm viability assay was carried out using the Live/Dead Sperm Viability kit (Thermo Fischer Scientific, Waltham, MA, USA). In brief, samples were firstly incubated with SYBR 14 dye (100 nM, 36 °C, 10 min) to examine live eupyrene sperm. Then, propidium iodide dye (12 μM, 36 °C, 10 min) was added to each sample to examine dead or damaged eupyrene sperm. Finally, samples were mounted on glass slides and imaged using a Leica SP8 confocal microscope (Leica Microsystems, Wetzlar, Germany). Fluorescence signals were quantified with ImageJ v1.52 (NIH, Bethesda, MD, USA).

### 2.7. Metabolome Analysis

At 180 min post-mating, spermatophore contents from successfully mated females (× ds*GFP*♂ or × ds*GmAGSP1*♂) were collected in sterile water and snap-frozen in liquid nitrogen. For metabolite extraction, 100 μL of thawed sample was mixed with 500 μL of methanol/acetonitrile (1:1, *v*/*v*) solution and shaken for 30 s. After 10 min of ultrasonication, samples were incubated at −20 °C for 60 min. After centrifugation at 12,000 rpm for 15 min at 4 °C, the supernatant (500 μL) was lyophilized (Labconco FreeZone, Kansas City, MO, USA). The dried residue was reconstituted in 160 μL of methanol/acetonitrile (1:1, *v*/*v*), vortexed for 30 s, sonicated for 2 min, and centrifuged again under identical conditions. Four biological replicates were processed, with 10 μL from each sample being pooled to generate a quality control (QC) sample.

Chromatographic separation was conducted using an Acquity I-Class PLUS UPLC system (Waters, Milford, MA, USA) coupled to a Xevo G2-XS QTOF mass spectrometer (Waters). The liquid chromatography–mass spectrometry (LC-MS) conditions were consistent with previously published methodology [22]. The raw LC-MS data were collected using MassLynx software (v4.2, Waters). Subsequently, these data were analyzed via Progenesis QI software (v2.0, Waters) by comparing them against both public databases and a self-built database (developed by Biomarker, Beijing, China). The normalized LC-MS data were subjected to in-depth analysis using R software (v 3.6.1). Specifically, principal component analysis (PCA) was executed using the prcomp function (v3.6.1), and orthogonal projections to latent structures discriminant analysis (OPLS-DA) was carried out with the ropls package (v1.6.2). Significance thresholds for differential abundant metabolites were defined as fold change > 1 (treatment vs. control), variable importance in the projection (VIP) > 1 from OPLS-DA models, and *p* value < 0.05. Volcano plot was used to filter metabolites by ggplot2 (v 3.3.0). Functional annotation of significant metabolites was conducted via Kyoto Encyclopedia of Genes and Genomes (KEGG) pathway mapping.

### 2.8. Data Analysis

All data were collected from at least three biological replicates and expressed as mean ± SD. GraphPad Prism 9.5.1 (GraphPad Software, La Jolla, CA, USA) was used for data analysis. Specific statistical tests are described in the figure legends.

## 3. Results

### 3.1. Characterization of GmAGSP1

**Figure 1:**
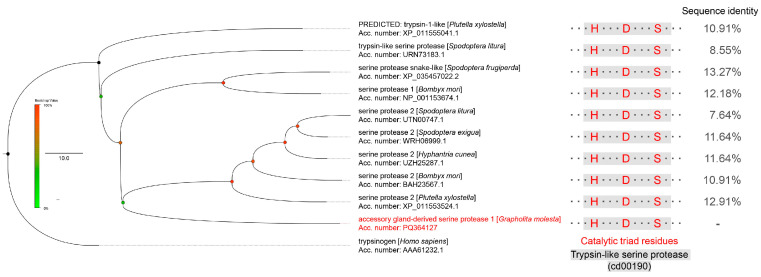
Phylogenetic tree and sequence analysis of GmAGSP1 protein with other identified sperm-activating SPs.

GmAGSP1 was identified through integrated transcriptomic and proteomic analyses and confirmed by sequencing. The sequence of GmAGSP1 contained a 310-amino acid (aa) open reading frame. Multiple sequence alignment with other identified sperm-activating SPs demonstrated that these proteins all contained the Tryp_SPc domain (accession: cd00190) and catalytic triad residues (H, D, and S) (Figure 1). This result showed that GmAGSP1 belongs to a typical SP family. Furthermore, phylogenetic analysis indicated that GmAGSP1 was only remotely related to those of other identified SPs (sequence identity from 7.64% to 13.27%) (Figure 1).

**Figure 2:**
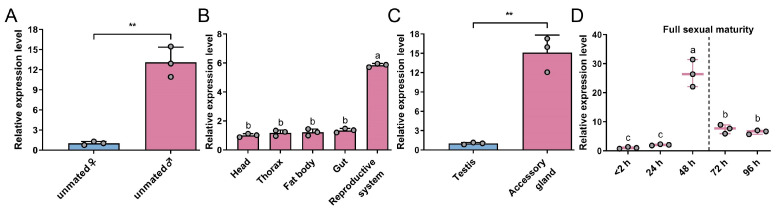
Gene expression profiles of *GmAGSP1* in adults. (**A**) Transcript distribution of *GmAGSP1* in unmated females and mated males. “Unmated ♀” expression was set as calibrator. (**B**) Expression pattern of *GmAGSP1* in male tissues. “Head” expression was set as calibrator. (**C**) Expression level of *GmAGSP1* in the testis and accessory gland. “Testis” expression was set as calibrator. (**D**) Expression of *GmAGSP1* in the accessory gland during the full sexual maturation. “<2 h” expression was set as calibrator. Data represent mean ± standard deviation (SD). Asterisks (**, *p* < 0.01, two-tailed Student’s *t*-test) and different lower case letters (*p* < 0.05, one-way ANOVA) indicate significant differences in relative expression.

Subsequently, *GmAGSP1* expression features were studied. The results indicated that *GmAGSP1* expression was male-biased, with significantly higher levels in males compared to females (Figure 2A). We then quantified the *GmAGSP1* mRNA levels in five major male tissues and observed that *GmAGSP1* was more highly expressed in the reproductive system (Figure 2B). Within the reproductive system, *GmAGSP1* was much more highly enriched in AG than testis (Figure 2C). With respect to the developmental stage, *GmAGSP1* was highly expressed at 48 h after emergence and then declined rapidly after full sexual maturity (Figure 2D).

### 3.2. GmAGSP1 Affects Male Fertility Without Influencing Sexual Behavior

**Figure 3:**
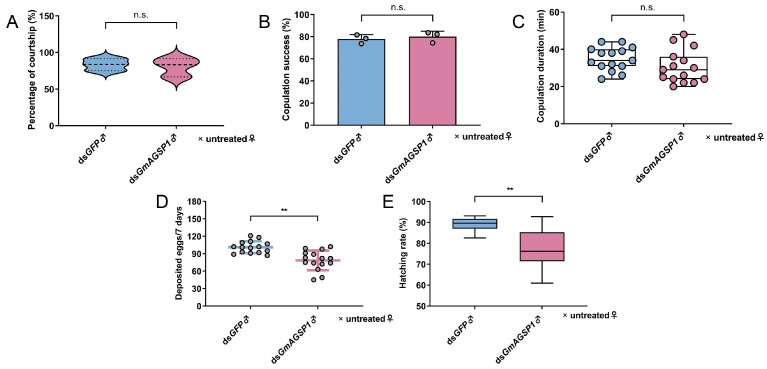
Effect of *GmAGSP1* knockdown on reproductive behavior and fertility in OFM males. (**A**) Analysis of percentage of courtship. (**B**) Analysis of proportion of successful mating. (**C**) Analysis of mating duration. (**D**) The number of eggs deposited in 7 days. (**E**) The accumulative hatching rate of laid eggs. Treatment group: ds*GmAGSP1*♂ × untreated♀; control group: ds*GFP*♂ × untreated♀. Data represent mean ± standard deviation (SD). n.s., *p* > 0.05; **, *p* < 0.01 (Fisher’s exact test for (**A**,**B**); Mann–Whitney *U* test for (**C**–**E**)).

The results of qRT-PCR indicated that *GmAGSP1* expression in the AG decreased by 51.23% at 48 h after treatment with *GmAGSP1* dsRNA compared to treatment with *GFP* dsRNA (Figure S1). Then, the effects of *GmAGSP1* knockdown on male sexual behavior were investigated. Knockdown of *GmAGSP1* did not significantly alter male courtship rate (treatment group, 80.53%, vs. control group, 83.47%) (Figure 3A), mating success (77.90% vs. 80.03%) (Figure 3B), or copulation duration (34.87 vs. 30.53 min) (Figure 3C) compared to controls. Furthermore, it was striking that the knockdown of *GmAGSP1* resulted in a significant change in male fertility: the total deposited egg number (control group, 101.20, vs. treatment group, 78.53) and the accumulative egg hatching rate (88.78% vs. 77.92%) were both significantly reduced (Figure 3D,E).

### 3.3. GmAGSP1 Promotes Eupyrene Sperm Maturation in the Spermatophore

**Figure 4:**
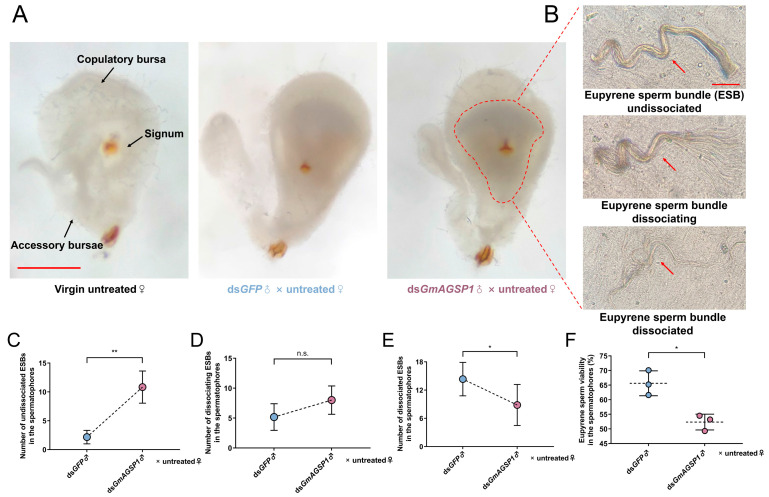
Effect of *GmAGSP1* knockdown on eupyrene sperm maturation in the spermatophores. (**A**) Images of bursa copulatrix and spermatophores after mating. Scale bar: 500 μm. (**B**) Images of eupyrene sperm bundles (ESBs) at different states isolated from spermatophores (ESBs were marked with red arrows). Scale bar: 50 μm. (**C**) The number of undissociated ESBs. (**D**) The number of dissociating ESBs. (**E**) The number of dissociated ESBs. (**F**) The viability of the individual eupyrene sperm. Treatment group: ds*GmAGSP1*♂ × untreated♀; control group: ds*GFP*♂ × untreated♀. Data represent mean ± standard deviation (SD). n.s., *p* > 0.05; *, *p* < 0.05; **, *p* < 0.01 (two-tailed Student’s *t*-test).

To explore the effects of *GmAGSP1* on eupyrene sperm maturation within the spermatophore, we studied the consequence of its knockdown on the dissociation state of ESBs and viability of individual eupyrene sperm. The morphological observation indicated that there was no significant difference in the BC between the treatment and control groups, and both groups form a balloon-like white spermatophore inside (Figure 4A). To accurately analyze the phenotype of ESBs, dissociation grades were first established. Figure 4B demonstrates that the dissociation grades of ESBs can be grouped into three stages: undissociated, dissociating, and dissociated. Based on this, we counted the number of ESBs in different dissociation states and found that the number of undissociated ESBs was significantly increased in the spermatophore of untreated♀ (× ds*GmAGSP1*♂) compared to those mated with ds*GFP*♂ (Figure 4C). No differences in the number of dissociating ESBs were detected between treatment group and control group (Figure 4D). In contrast, the number of dissociated ESBs was dramatically decreased in the treatment group compared to the control group (Figure 4E). Finally, the activities of individual eupyrene sperm after treatment with dsRNAs were measured. In the spermatophore, the proportion of inactive eupyrene sperm in the control group (mated with ds*GFP*♂) decreased to 34.37%, compared to 47.67% in the treatment group (mated with ds*GmAGSP1*♂) (Figure 4F).

### 3.4. Metabolomic Analysis in the Spermatophore of Females upon GmAGSP1 Knockdown

**Figure 5:**
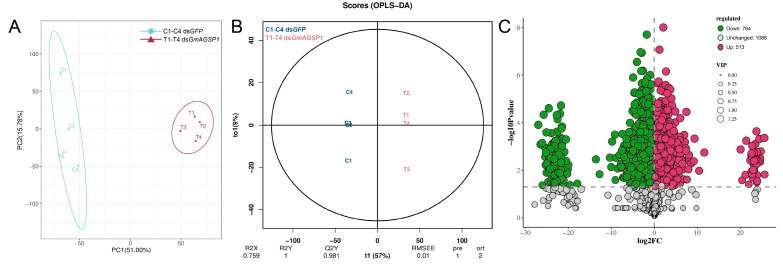
PCA score plot (**A**), OPLS-DA score plot (**B**), and volcano plot (**C**) of metabolic profiles of spermatophores from the control (ds*GFP*♂ × untreated♀) and treatment (ds*GmAGSP1*♂ × untreated♀) groups.

PCA and OPLS-DA models were employed to analyze the metabolic profiles of spermatophores in the control group (ds*GFP*♂ × untreated♀) and the treatment group (ds*GmAGSP1*♂ × untreated♀). As shown in Figure 5A,B, both PCA and OPLS-DA score plots revealed distinct metabolite differences between the two groups. A total of 1277 bioactive compounds were annotated as differentially abundant metabolites. Among these differentially abundant metabolites, 513 compounds were upregulated when comparing the control group with the treatment group, while 764 compounds were downregulated (Figure 5C).

**Figure 6:**
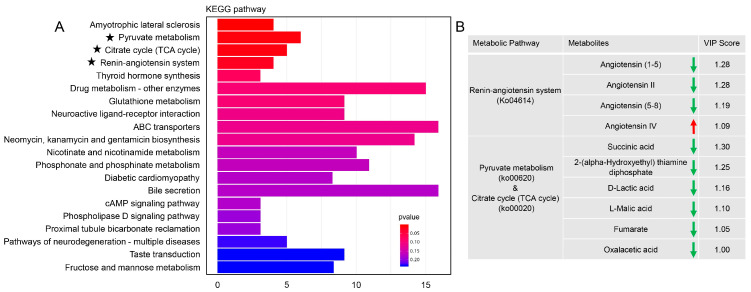
(**A**) Bar plot of differential metabolite pathways in each group (ds*GFP*♂ × untreated♀ vs. ds*GmAGSP1*♂ × untreated♀). (**B**) VIP scores of identified metabolites and associated metabolic pathways. The asterisk represents the three metabolic pathways analysed in Figure 6B.

To further characterize the biochemical changes, a bar plot was constructed using the KEGG metabolic pathway database. Metabolites that were differentially abundant between treatment and control groups were enriched in multiple metabolic pathways. Based on the differential abundance scores, “amyotrophic lateral sclerosis”, “pyruvate metabolism”, “citrate cycle (TCA cycle)”, “renin-angiotensin system”, and “thyroid hormone synthesis” were the top five pathways (Figure 6A). Given the established association of “amyotrophic lateral sclerosis” and “thyroid hormone synthesis” with human diseases, these pathways are unlikely to directly account for the observed phenotype. In addition, VIP scores showed that, except for angiotensin IV in the “renin-angiotensin system”, other metabolites in the “renin-angiotensin system”, “pyruvate metabolism”, and “citrate cycle (TCA cycle)” pathways were dramatically downregulated (Figure 6B).

## 4. Discussion

Seminal fluid proteins (SFPs), transferred from males to females during mating, play a key role in ensuring reproductive success across diverse taxonomic groups, including *Drosophila melanogaster* [23], *Culex pipiens* [24], *Allonemobius socius* [25], and *B. mori* [11]. While transcriptomic studies identified that “endopeptidase activity” is essential for sperm activation [17], the specific genes have not been identified and characterized. Therefore, this study mainly focused on the sequence characteristics, expression patterns, functional characterization, and global metabolomic profiling of a sperm-activating SP in *G. molesta*.

Expression profiling revealed that *GmAGSP1* was most abundant in the male AG during the pre-sexual maturity stage, in accordance with identified SPs in other insects, such as *S. litura* [14], *Spodoptera exigua* [8], and *B. mori* [6]. They all exhibit the typical expression characteristics of ACPs. However, phylogenetic analysis of GmAGSP1 revealed remarkably divergent primary sequence compared to the well-characterized serine protease 2 (BmSer2) in *B. mori* and other annotated sperm activation-related SPs. Therefore, it is highly likely that these SPs transferred to the spermatophores after mating exhibit significant differences in both catalytic activity and substrate specificity. This evolutionary adaptation implies that sperm activation requires a tightly coordinated proteolytic cascade, mechanistically analogous to the hemostatic cascades in mammalian systems [26]. Notably, this biochemical network relies on sequential activation of multiple proteases with strict temporal regulation. Future investigations will focus on functional purification of bioactive GmAGSP1 from secretory tissues, coupled with LC-MS-based degradomic profiling to elucidate its upstream activators and downstream proteolytic targets within this signaling cascade.

The Sterile Insect Technique (SIT), including radiation-based sterile technology (rSIT) and genetic-based inheritable sterile technology (gSIT), has become an effective method for controlling pest populations [6]. A recent study in *S. litura* demonstrated that gamma irradiation significantly downregulated the expression of AG-derived sperm-activating protease in both males and their F_1_ offspring [14]. This finding suggests that disruption of genes encoding these proteases plays a critical role in the efficacy of the F_1_ sterility technique, potentially by impairing sperm activation and subsequent reproductive success [14]. Furthermore, another AG-derived sperm-activating protease, Ser2, specifically modulated male fertility but did not affect female fertility and did not impact growth and development in *B. mori* [6], *Plutella xylostella* [6], *S. litura* [7], *S. exigua* [8], and *Hyphantria cunea* [9], making it an ideal target for population control based on gSIT. Similarly, in this study, RNAi-mediated knockdown of *GmAGSP1* resulted in substantially reduced male fertility, whereas the courtship rate, copulation duration, and mating rate remained unaffected. This suggests that targeting sperm activation regulatory genes in gSIT is both feasible and operationally viable for practical applications.

The spermatophore, a unique post-mating structure formed in lepidopteran insects, serves as a dynamic microenvironment orchestrating intense physiological and biochemical reactions [13]. While studies in *B. mori* [11], *S. frugiperda* [13], and our work in *G. molesta* have all demonstrated the regulatory role of SPs in eupyrene sperm activation, the global metabolic profiling of spermatophores during critical time points remains unexplored. In this study, we utilized untargeted metabolomics to explore the metabolic features and overall profiles of the spermatophore following the knockdown of *GmAGSP1*. Our findings revealed that numerous metabolic pathways underwent significant alterations. Among these, the “pyruvate metabolism”, “citrate cycle” and “renin-angiotensin system” pathways were the most prominently affected. Previous studies have shown that glycolysis (from glycogen to pyruvate) and modified citrate cycle (from phosphoenolpyruvate and from 2-oxoglutarate to succinate) are involved in sperm activation [15,27]. These findings suggest that glycolysis and the citrate cycle may represent conserved metabolic modules supporting sperm activation in Lepidoptera, though further comparative studies across species are needed to confirm their ubiquity. Another significantly affected pathway is “renin-angiotensin system”. The renin-angiotensin system (RAS) is a peptidic signaling network exhibiting endocrine properties, predominantly recognized for its pivotal role in blood pressure regulation [28]. In mammals, multiple components of this system have been extensively characterized across various tissues of the male reproductive tract, including the prostate, epididymis, and ductus deferens [29]. Especially, accumulating data have indicated that angiotensin II (Ang II) is involved in sperm motility, capacitation, and sperm–egg fusion [30,31]. In insects, only a few studies have reported that angiotensin converting enzyme (hydrolyzes Ang I to Ang II) is associated with sperm activation [32]. However, systemic regulatory mechanisms of RAS in sperm activation and other post-mating responses remain unstudied and represent a critical gap for future work.

## 5. Conclusions

We characterized a sperm-activating protease (GmAGSP1) in the OFM, and elucidated its critical role in male reproductive biology and metabolic regulation. Given the polygamous mating behavior of OFM males, investigating male-derived sperm activators offers promising targets like GmGSP1, which could enhance OFM population control strategies.

## Data Availability

The data that support the findings of this study are available from the corresponding author upon reasonable request.

## Supplementary Materials

The following supporting information can be downloaded at: https://www.mdpi.com/article/10.3390/insects16080782/s1, Table S1: Primers used in this study; Figure S1: Effect of dsRNA injected into newly emerged adults on the gene transcript levels of OFM. Knockdown efficiency was measured at 48 h after ds*GmAGSP1* injection. ds*GFP* was used as control. Data represent mean ± standard deviation (SD). **, *p* < 0.01 (two-tailed Student’s *t*-test).
