# Supplementary material for: Maturation of Eupyrene Sperm upon Ejaculation Is Influenced by a Male Accessory Gland-Derived Serine Protease in Grapholita molesta
Source: Insects. 2025 Jul 30;16(8):782. doi: 10.3390/insects16080782 (PMC12386725; doi:10.3390/insects16080782)
Supplement: Supplementary file 1 [file insects-16-00782-s001.zip › insects-3726511-supplementary.pdf]

Supplementary Material

Table S1 Primers used in this study

Gene	Direction	Sequence (5′-3′)
cDNA cloning		
GmAGSP1	F	ATGGTGAATCTTCTTAGATC
	R	TCAAGTTGTCATTATGTTGT
qRT-PCR		
GmAGSP1	F	GAGGTGGATTGGATGTGTGG
	R	GGGTCTGTCTCCGTGTTGTG
Actin	F	CTTTCACCACCACCGCTG
	R	CGCAAGATTCCATACCCA
GAPDH	F	GGAAAGCTGACTGGTATGG
	R	ACCTGGTCCTCGGTGTAG
RNA interference		
dsRNA-AGSP1	F	taatacgactcactatagggGCCGAAAGTCATCAAACCTC
	R	taatacgactcactatagggGAAGGAAACGACCCCGAT
dsGFP	F	taatacgactcactatagggCAGTTCTTGTTGAATTAGATG
	R	taatacgactcactatagggTTTGGTTTGTCTCCCATGATG

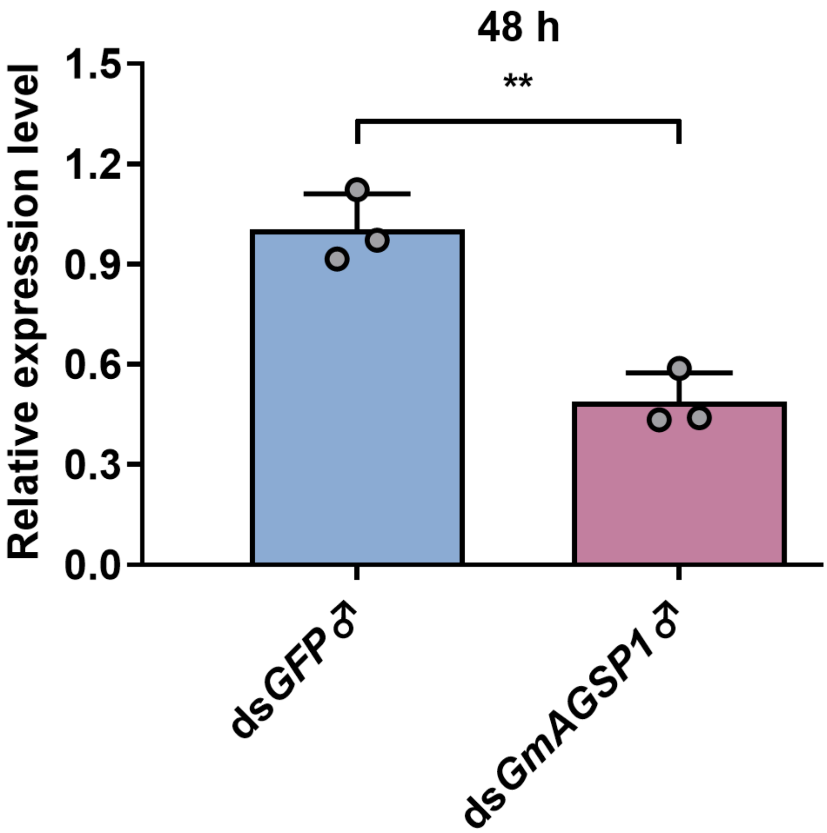

Figure S1. Effect of dsRNA injected into newly emerged adults on the gene transcript levels of OFM. Knockdown efficiency was measured at 48 h after ds*GmAGSP1* injection. ds*GFP* was used as control. Data represent mean ± standard deviation (SD). **, $p < 0.01$ (two-tailed Student's t-test).
